# Syllable as a Synchronization Mechanism That Makes Human Speech Possible

**DOI:** 10.3390/brainsci15010033

**Published:** 2024-12-30

**Authors:** Yi Xu

**Affiliations:** Department of Speech, Hearing and Phonetic Sciences, Division of Psychology and Language Sciences, University College London, Chandler House 2 Wakefield Street, London WC1N 1PF, UK; yi.xu@ucl.ac.uk

**Keywords:** speech production, articulation, speech motor control, syllable, segmentation, resyllabification, target approximation, synchronization

## Abstract

Speech is a highly skilled motor activity that shares a core problem with other motor skills: how to reduce the massive degrees of freedom (DOF) to the extent that the central nervous control and learning of complex motor movements become possible. It is hypothesized in this paper that a key solution to the DOF problem is to eliminate most of the temporal degrees of freedom by synchronizing concurrent movements, and that this is performed in speech through the syllable—a mechanism that synchronizes consonantal, vocalic, and laryngeal gestures. Under this hypothesis, syllable articulation is enabled by three basic mechanisms: target approximation, edge-synchronization, and tactile anchoring. This synchronization theory of the syllable also offers a coherent account of coarticulation, as it explicates how various coarticulation-related phenomena, including coarticulation resistance, locus, locus equation, diphone, etc., are byproducts of syllable formation. It also provides a theoretical basis for understanding how suprasegmental events such as tone, intonation, phonation, etc., are aligned to segmental events in speech. It may also have implications for understanding vocal learning, speech disorders, and motor control in general.

## 1. Introduction

Although nearly everyone can identify syllables, almost nobody can define them.

—Ladefoged (1982, p. 220) [1]

The human motor apparatus … comprises more than 200 bones, 110 joints and over 600 muscles, each one of which either spans one, two or even three joints. While the degrees of freedom are already vast on the biomechanical level of description, their number becomes dazzling when going into neural space.

—Huys (2010, p. 70) [2]

One of the fundamental problems in neuromotor control, as first recognized by Nikolai Bernstein [3], is that of too many degrees of freedom (DOF). That is, most motor movements involve multiple body structures, so that it would be immensely difficult for the central nervous system to control them separately. Speech, also as a motor skill, faces the same problem. To say a simple syllable like [ma], for example, multiple articulatory gestures need to be made concurrently: closing the glottis and increasing the lung pressure to generate voice, closing the lips and lowering the velum to produce the nasal sound [m], lowering the jaw, lowering and retracting the tongue body to produce the vowel [a] [4]. And, if the [ma] is said with a lexical tone in a language like Mandarin, the vocal folds have to be adjusted to raise or lower F_0_, often more than once, within the same syllable [5]. All of these need to be completed in less than one-fifth of a second [6]. In fact, it would be hard to imagine how any motor action involving more than one body structure and/or muscle would be possible without a solution to this DOF problem, and how speech would be possible without a means to reduce DOF to the extent that multiple articulators and the muscles driving them can be effectively controlled.

This DOF problem, also known as the Bernstein problem, may emerge at any level of neuromotor control, and there have been many proposals in the motor control literature on how to resolve it [7]. Bernstein’s own proposal is that motor redundancy can be minimized by freezing many of the degrees of freedom by organizing a group of muscles into a functional unit called *synergy*, “such that a central control signal jointly and proportionally activates all muscles in the synergy” (see p. 278 [7]). A further idea is that groups of muscles may form *coordinative structures* that act together to perform a single action [8,9]. Coordinative structure has also been adopted in some theories of speech production [10,11]. In particular, it is applied in the task dynamic (TD) model of speech articulation [11], which forms the computational basis of articulatory phonology (AP) [12]. In this TD/AP framework, articulatory gestures are assumed to form autonomous coordinative structures, which are then temporally overlapped with each other. To coordinate these overlapping gestures, a coupled oscillator model of timing planning is then applied [13,14,15]. In this model, each gesture is associated with an internal planning oscillator responsible for controlling the temporal pattern of its movement. In this conceptualization, however, not only the multiple gestures, but also their relative timing, are separately controlled. This would introduce *more* rather than less degrees of freedom.

The coupled oscillator model has been used to account for various synchronization phenomena in motor movements [16,17,18] because motor synchrony appears to resemble the well-known physical entrainment [19], as both show the shared timing of two oscillating movements. Entrainment is a physical phenomenon whereby two oscillating systems with similar natural frequencies, e.g., two pendulum clocks, gradually fall into synchrony when they are connected through some mechanical link, such as being hung on the same beam [19,20]. If coupled oscillation does not resolve the DOF problem as pointed out above, however, we may wonder whether entrainment is the right analogy for motor synchrony. Indeed, a careful comparison reveals that motor synchrony differs from entrainment in a number of critical ways, as listed in Table 1. First, motor synchrony can occur in bi-manual actions with no repeating cycles [21]. Such non-repeating synchrony, by definition, would be irrelevant to entrainment. But it is highly relevant for monosyllabic words spoken in isolation. Likewise, in speaking in unison—a skill surprisingly natural to most people without much practice, speakers can easily synchronize their reading aloud of the same text [22]. The non-periodic articulatory movements in speaking in unison cannot be accounted for by theories that use periodicity as the basis for explaining synchronization [23].

Second, in entrainment, it takes many cycles for two oscillators to reach synchrony. In motor synchrony, the shift from 180° (anti-phase) to 0° (in-phase) occurs in only 1–2 cycles [24,25,26,27], which is virtually instantaneous. In a system of coupled oscillators, the fastest phase shift [16] simulated with an oscillation model takes 5–6 cycles to complete. A gradual shift across 5–6 cycles also means that in some of those cycles, the phase relation is neither 180° nor 0°, which is exactly what has been repeatedly shown to be impossible in motor synchrony [26,27,28]. Furthermore, in coupled oscillation, each oscillator has to have its own initial phase condition. For speech, one would naturally ask, where do the initial phase conditions come from in the first place?

Third, as shown in the third and fourth rows of Table 1, entrainment requires that the synchronized oscillators are similar in their natural frequencies, and even after reaching synchrony, they may go out of phase again [29,30]. Neither high similarity in frequency nor phase instability is characteristic of motor synchrony [21,26]. 

Finally, probably the most fundamental difference is that in entrainment, the systems being synchronized are independent of each other, with no central control, and the synchrony is achieved passively through physical links between the involved systems [20]. Motor synchrony, in contrast, occurs between movements that are under a common central control, or in the case of synchrony between two individuals, under a shared control maintained through sensory monitoring [27]. The central control, as well as the sensory monitoring that makes it possible, is clearly lacking in physical entrainment.

The Bernstein problem therefore is unlikely to be solved by a coordinative structure constructed as a system of coupled oscillators [13,14,15]. To start with, there is no explicit scheme to reduce degrees of freedom. Rather, each of the assumed planning oscillators has to have its own natural frequency unrelated to the natural frequency of the associated gesture, and its own initial phase condition, which results in at least two additional degrees of freedom. Furthermore, entrainment is used to model both abrupt shifts from VC to CV in accelerating repetitive syllable sequences [25], and the planning of intergestural coordination is postulated to occur before the onset of each and every syllable [13,14,15]. It is especially problematic that it takes at least several cycles to complete a phase shift or to stabilize the phase relation during planning when they are computationally modeled as an entrainment process [16].

A viable alternative solution therefore needs to explicate how degrees of freedom can be effectively reduced. The solution considered in this paper is that for speech, the syllable serves exactly this function. This solution, however, suggests a theory of the syllable that differs from all past syllable theories. And it also touches on the long-standing issue of coarticulation. The following Section briefly reviews the state of the art for both syllable and coarticulation.

## 2. Syllable and Coarticulation

That speech utterances are made up of syllables may seem obvious. Most, if not all, early writing systems (Sumerian, Linear B, Akkadian cuneiform, Chinese, Mayan, etc.) started as syllabaries, in which the written symbols represent syllables (or sometimes morae) rather than consonants and vowels [31,32,33]. It is also much easier for anyone, including non-experts, to count the number of syllables in a word than the number of segments in a syllable [33,34,35]. The syllable is also known to play many important roles in speech. It is the unit that carries stress and accent [36,37,38], rhythm [39,40,41], and tone [42,43]. It is said to be a unit in a prosodic hierarchy of constituents [44,45], which influences patterns of segmental allophony, and a unit that serves as the domain of applying language-specific phonotactic patterns and other phonological rules [46,47]. It is also critical for the perceptual segmentation of the speech signal [48,49,50]. However, neither our intuition about it nor its own usefulness has been sufficient to avert doubts about the syllable. After examining eight lines of traditional evidence in support of the syllable as a representation unit in speech production, Shattuck-Hufnagel in 2011 found none of them unequivocal [35]. The lack of clear evidence has led to skepticism about its existence [51] or universality [52]. Similar reservations have been expressed by Gimson [53], Steriade [54], and Blevins [55]. A major reason behind these doubts is that the nature of the syllable has remained vague. In particular, no theory has been able to address some of the hardest questions:

- Why are there syllables?
- Do syllables have clear phonetic boundaries?
- Do segments have definitive syllable affiliations?

### 2.1. Why Are There Syllables?

For any scientific inquiry, the *why* question is often the most essential yet the most difficult. This is also the case with the syllable. The functional use of the syllable, as mentioned above, to carry stress, accent, tone, rhythm, or to serve as a domain of phonological rules, are all extended benefits of the syllable, and so cannot be the primary reasons for the existence of the syllable in the first place. What we are seeking is an account of why the syllable is indispensable, i.e., serving a function that is so vital that speech would be impossible without it.

Some theories have taken the syllable as the basic unit of speech, e.g., Stetson’s motor phonetics [56] and Fujimura’s C/D model [57]. But they have offered no explicit proposal as to why syllables are obligatory at the articulatory level. In MacNeilage’s frame/content theory [58], the syllable is suggested to have evolved from the oscillation of the jaw in movements like chewing, sucking, and licking. However, the ability to oscillate the jaw is shared by virtually all mammals, yet not even our closest relatives, i.e., chimpanzees, gorillas, and bonobos, have developed syllable-based speech [59,60]. Thus, being able to oscillate the jaw does not seem to inevitably lead to an ability to articulate syllables. Something extra must be involved. 

It has also been proposed that the syllable is a unit of stored motor programs [61,62]. But the proposal is questioned for its inability to explain cases of resyllabification or the lack thereof [35]. More importantly, even if stored syllable-sized motor programming is shown to exist, it cannot explain why the unit has to have the form of a syllable. It thus remains an open question as to whether the syllable, with its own unique characteristics, is indispensable, i.e., serving a function that is so vital that speech would be impossible without it.

### 2.2. Are There Clear Boundaries to the Syllable?

**Figure 1:**
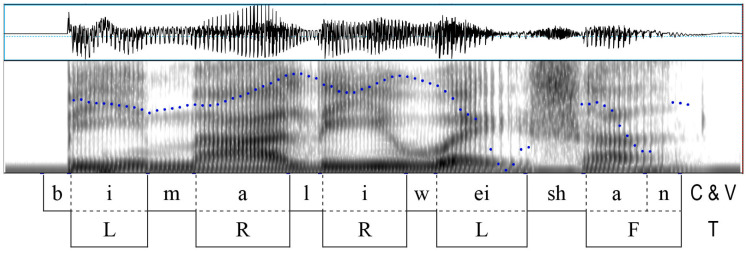
Spectrogram of the Mandarin phrase “比麻黎偽善” /bǐ má lí wěi shàn/ [more hypocritical than Ma Li], with broad phonetic transcriptions and pitch tract (in blue). C, V, and T stand for consonant, vowel, and tone, respectively. The segmentation is based on well-known conventions [63,64]. The segmentation of /w/ is based on Peterson and Lehiste (1960) [65].

Given an utterance like the one shown in Figure 1, it may seem that some of the syllables are well separated by the alternation of consonants and vowels whose spectral patterns show clear boundaries [63]. However, the syllable boundaries are much less clear-cut in the case of /wei/. Because it begins with a glide /w/, it is hard to determine when the preceding syllable ends and the next one starts. Even more difficult are cases where a word starts with a vowel, as in the English words like *artist*, *article*, *articulate,* and *arbitrary*. When they are preceded by words ending in a vowel, as in *new artist*, *my article*, *to articulate*, or *fairly arbitrary*, there would be continuous formant movements across the word (hence, syllable) boundaries (unless when spoken very carefully so that the syllable starts with a glottal stop). The same problem would be seen in cases of word-internal syllables, like in *hiatus*, *appreciate*, *mediocre*, etc., where there should presumably be a syllable boundary between /i/ and the following vowel or diphthong, yet all we can see in the spectrogram in most cases are continuous formants between the preceding and following consonants.

The difficulty of syllable boundary identification has led to the view that it is simply futile to look for clear-cut boundaries in the speech signal, as argued by Hockett [66], who likens segments to colored raw Easter eggs lined up on a belt. After being crushed by a wringer, the heavy smearing makes the edges of the individual eggs unrecognizable. The problem with this analogy is, however, if we do not know where the boundaries are, how can we be so certain that segments are heavily overlapped with each other [67,68]? So, the fuzziness of the syllable boundaries is directly related to the fuzziness of segmental boundaries, which in turn is related to yet another major conundrum of human speech: coarticulation, as will be discussed in Section 2.4. 

### 2.3. Do Segments Have Definitive Syllable Affiliations?

The clarity of syllable boundaries hinges on not only the clarity of segmental boundaries, but also the certainty about where each and every segment should belong in a syllable: onset, offset, or between two adjacent syllables, i.e., being ambisyllabic. There have been many theories of syllabification, including the law of initials and the law of finals [69], the maximal onset theory [70,71], the theory that stressed syllables are maximized [72,73], and the weight-stress principle [73,74,75]. But so far, there has been no consensus on even some of the simplest cases. For the word *happy*, for example, at least four ways of syllabification are possible as summarized by Duanmu [76]: /hæ.pi/, /hæp.i/, /hæpi/, and /hæp.pi/ (where the period stands for syllable boundary and an underscore indicates the segment is ambisyllabic). All these syllabification theories, however, are based on intuition or non-experimental phonological analyses. There are also experimental investigations of syllabification intuition by naïve speakers [49,77,78,79]. None of the syllabification findings, however, has directly addressed the issue of what syllable boundaries look like in the acoustic signal or in terms of articulatory movements. 

### 2.4. Are Syllables Related to Coarticulation?

The problems of syllable boundary and syllable affiliation of segments discussed above are both closely related to another long-standing problem, namely, coarticulation. The term coarticulation, initially “Koartikulation” in German, was coined to refer to articulatory timing around syllable onset [80]. The observation was that “the articulatory movements for the vowel in tokens such as /ma/ or /pu/ began at the same time as the movements for the initial consonant” ([68] p. 14). The link between syllable and coarticulation is further strengthened by Kozhevnikov and Chistovich [81], who proposed the notion of *articulatory syllable*, based on the observation that in Russian, the lip protrusion of /u/ begins at the same time as the first consonant in a consonant cluster. According to this notion, the domain of coarticulation is the articulatory syllable, in the sense that all the articulatory actions connected with the articulatory syllable, including the vowel, start at the syllable onset, as long as the consonantal movements do not contradict the articulation of the vowel.

The articulatory syllable, however, has been questioned due to uncertainties over the temporal scope of vowels in a syllable [82,83,84,85]. A major reason for the skepticism is the widely reported preparatory activities, particularly the classic finding of Öhman [86], and the phenomenon of vowel harmony [87], which seem to suggest that the scope of the vowel extends well before the syllable onset. By now, the term coarticulation is generally used to refer to virtually any variability of a segment due to the influence of surrounding segments [68].

Perhaps the most detailed account so far of coarticulation as related to the syllable is offered by the TD/AP framework [13,14], as mentioned earlier. In the most recent version of TD/AP, syllable structure is modeled as emerging from coupled oscillations as internal planners that are in-phase between consonant and vowel gestures at the syllable onset but anti-phase at the syllable offset. The in-phase coupling of CV at the syllable onset is consistent with the notion of articulatory syllable [20], and accounts for a large amount of CV coarticulation. However, the TD/AP account of the syllable leaves some core problems unresolved. The most critical is the assumption that each gesture is controlled by a planning oscillator whose frequency and initial phase both need to be specified, making it unclear how DOF can be effectively reduced. Second, the in-phase and anti-phase assumption for syllable onset vs. offset is based on empirical observations, but there is no account of why the asymmetry is there in the first place. Finally, as shown in Table 1, it is questionable that coupled oscillation based on physical entrainment is the right model for motor synchrony due to multiple discrepancies between the two phenomena.

## 3. Syllable as a Synchronization Mechanism

**Figure 2:**
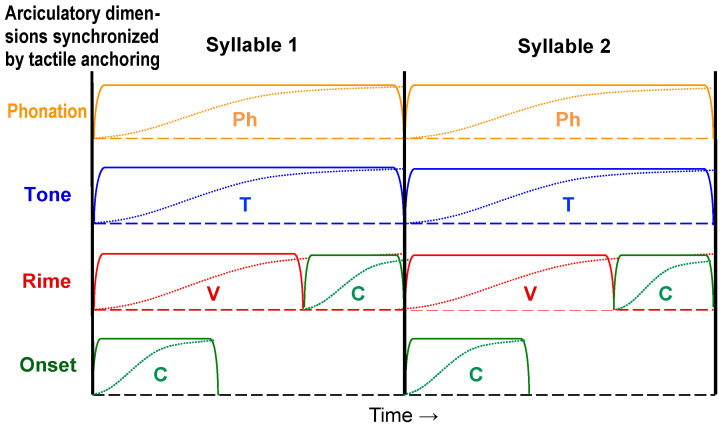
Temporal organization of articulatory dimensions under the synchronization hypothesis. The tiers represent the four articulatory domains controlled by the central nervous system: C for conant, V for vowel, T for tone, and Ph for phonation. The dotted curves represent asymptotic articulatory approximation of underlying targets (*target approximation*). The full alignment of the onsets and offsets of the approximation movements represent *edge synchronization* facilitated by *tactile anchoring*.

The hypothesis considered in this paper is that the DOF problem is solved by actively controlled motor synchrony, which, in the case of speech, is achieved through the syllable. This synchronization fixes the relative timing of multiple motor movements so that most of the temporal degrees of freedom are eliminated, not only in learning, but also in normal operation (as opposed to Bernstein’s proposal that the freezing of DOF is mainly for learning). For speech, the formation of the syllable is also the mechanism underlying coarticulation. The syllable model based on this hypothesis consists of three core mechanisms: *target approximation*, *edge synchronization*, and *tactile anchoring*, as sketched in Figure 2. Target approximation (the dashed curves) is the articulatory process of executing phonetic targets, and it is what defines the articulatory gesture (see Section 3.1 for details). Edge synchronization (indicated by the vertical lines) is the mechanism of coordinating multiple gestures that make up a syllable, including consonant, vowel, tone, and phonation. (Here, phonation refers to the use of voice quality as an independent dimension to mark lexical contrasts, which is found in some languages [88,89]. It does not refer to phonation properties that accompany consonant manner of articulation). And tactile anchoring (not directly represented in Figure 2) is the facilitation of edge synchronization by sensory feedback, mainly through tactile sensation during consonant closures.

**Figure 3:**
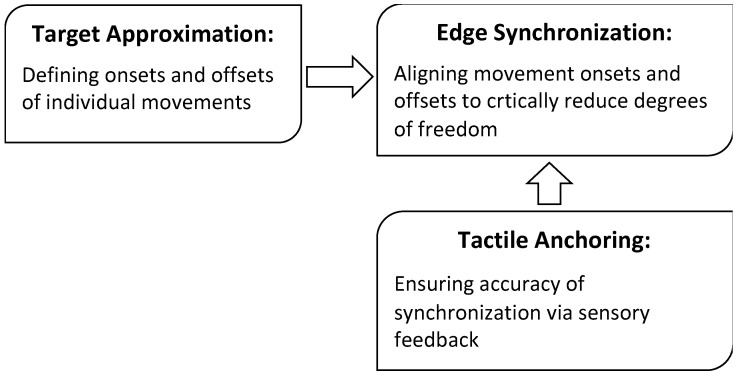
Interdependence of the three mechanisms of the synchronization hypothesis. Target approximation defines the onsets and offsets of individual movements; edge synchronization aligns movement onsets and offsets (rather than acoustic landmarks); and tactile anchoring provides the sensory feedback that ensures the accuracy of synchronization.

Conceptually, the three mechanisms of the synchronization hypothesis are interlocked as illustrated in Figure 3: target approximation is what defines the onsets and offsets of individual movements; movement onsets and offsets (not acoustic landmarks) are what edge synchronization aligns; and tactile anchoring is what ensures the accuracy of synchronization.

By positing the syllable as a mechanism for solving the DOF problem, the synchronization hypothesis not only offers an account of the syllables that deviate from existing theories but also provides an account of coarticulation, as will be detailed in Section 3.2.1, Section 3.2.2 and Section 3.2.3. In the following sections, each of the three core mechanisms of the synchronization hypothesis will be elaborated, with support from existing literature. Also discussed will be the similarities and differences between this hypothesis and other models in addressing various specific aspects of the syllable. Finally, Section 4 and Section 5 will present new evidence obtained in the latest studies.

### 3.1. Targets and Target Approximation

**Figure 4:**
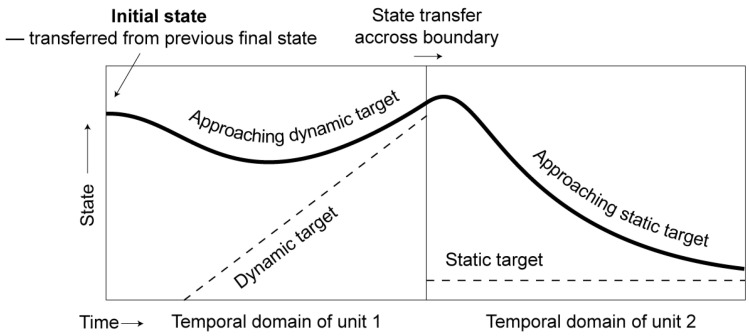
The target approximation model. A schematic illustration of hypothetical phonetic targets (dashed lines) and their surface realization (solid curve). The three vertical lines represent the boundaries of the two consecutive target intervals. The level dashed line on the right represents a static target, and the oblique dashed line on the left represents a dynamic target. In both intervals, the targets are asymptotically approximated. Adapted from the original version for tone and intonation [92].

The notion of *target approximation* goes back at least as far as Lindblom (1963) [90], who suggests that underlying phonetic targets are often only partially realized due to time constraints. Similar ideas are shared by a number of models proposed since Lindblom [90], in particular, the Fujisaki model of intonation [91], and the TD/AP framework for segmental articulation [11,12]. The version of target approximation presented here [92], as schematized in Figure 4, was independently developed based on empirical data on contextual tonal variations [5,93,94,95]. 

In this model, each movement is a process of approaching an underlying target (dashed lines in Figure 4) within an extrinsically designated temporal interval. Each target approximation movement is controlled by three parameters: target position, target slope (underlying velocity), and target strength. Adjacent target approximations are *contiguous without overlap*, shifting abruptly from one to the next at each interval boundary. The resulting surface contour (solid curve in Figure 4) is nevertheless smooth and continuous due to the transfer of dynamic states at the boundary. Despite similarities with other models, there are five key properties that are unique to the target approximation model presented in Figure 4:

1. Surface acoustic forms result from asymptotic approximation of a *single* sequence of underlying targets rather than from superposition of multiple underlying contours [91,96,97].
2. Targets are approximated *sequentially*, with neither overlap of adjacent movements along the same articulatory dimension [98], nor gaps in between [38,91], unless there is a silent pause. The lack of gaps also means that there are no temporal intervals (except pauses) without targets.
3. Targets can be *intrinsically dynamic*, i.e., with underlying slopes of various degrees. No other model, to our knowledge, has incorporated dynamic targets. (See Section 3.1.5 for critical differences between underlying velocity and surface velocity. The former is a property of the target, which can be either static or dynamic, while the latter is the consequence of executing the target. Some models, like TD and Fujisaki models, specify the stiffness of the target gesture, which indirectly specifies surface velocity. But they have no specifications for underlying velocity. So, a fully achieved target in those models can only generate an asymptote to a static articulatory state.)
4. Every target also has a strength specification, which determines the rate at which the articulatory goal is approached. Target strength (or stiffness) is treated in other models as either mostly fixed [91,98] or a means of controlling duration [99].
5. Target duration is not predominantly determined by the time needed to reach the target, or intrinsic timing [12,100], but by functional factors such as lexical contrast (lexical quantity, lexical tone, and lexical stress), focus, and boundary marking [101].

The target approximation model has been quantified in the form of qTA [102], which has been applied to English, Mandarin, Thai, Japanese, Arabic, Persian, Savosavo, Fijian, and Vietnamese [103,104,105,106,107,108,109,110,111,112]. The present paper will not focus on the quantitative aspect of the model, but some of the graphics are generated with qTA. Also, although the target approximation model was initially developed for tone and intonation, its relevance for segments has also been demonstrated [113,114,115]. Birkholz et al. [116] have developed a higher-order version of the target approximation model for an articulatory synthesizer, which has been successfully implemented in articulatory synthesis for English, Thai, and German [117,118,119,120,121].

In the following Section, the main properties of target approximation will be elaborated and evidence from the literature, wherever available, will be presented.

#### 3.1.1. Asymptotic Approximation

**Figure 5:**
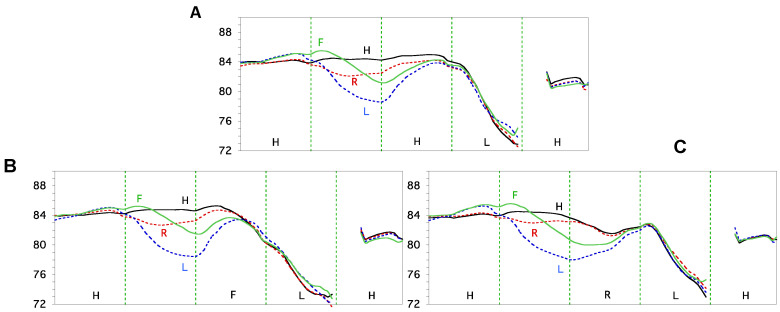
Mean time-normalized F_0_ contours of Mandarin tones in 5-syllable sentences, where all syllables are in the form of nasal + vowel. In each plot, the tones of all the syllables remain constant except those of the 2nd syllable, which alternate from High (H) to Rising (R), Low (L), and Falling (F). The tone of the third syllable varies from H in (**A**), F in (**B**), to R in (**C**). Data from Xu [94].

Among the clearest evidence of asymptotic approximation are the F_0_ trajectories of lexical tones found in connected speech [5,94]. As shown in Figure 5, the F_0_ of the tone in the third syllable in each plot starts at very different heights depending on the tone of the second syllable. Yet all the trajectories quickly accelerate away from the initial states, and converge, within the third syllable, to a linear configuration that reflects the tone’s underlying targets: high-level, falling, and rising, respectively. Similar asymptotic approximation has also been observed for vowels [122].

#### 3.1.2. Sequentiality

**Figure 6:**
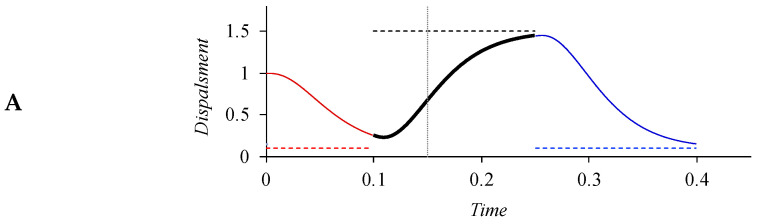

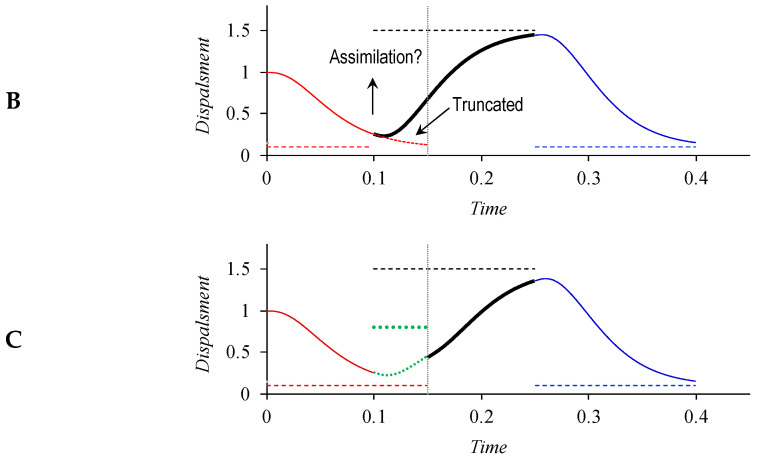
Sequential and blended target approximation processes generated with the qTA model [102]. The units of both axes are arbitrary. In (**A**), the three target approximation movements are strictly sequential, and the vertical line is the boundary between the first two movements. In (**B**), the vertical reference remains at time 0.15, but the first movement is shortened by 0.05 units. All the movements remain sequential (which means that the “tail” of the first movement—the dotted continuation—is truncated). In (**C**), the first and second movements overlap with each other by 0.05 units. The overlap is implemented by inserting a blended target (dotted green horizontal line), which is the average of the first two targets.

In Figure 4, although the surface trajectory is smooth and continuous, the underlying targets are strictly sequential, with neither gap nor overlap around the boundary. Thus, there is no need for specifications (*hence*, *no extra degrees of freedom*) on how much adjacent targets along the same articulatory dimension overlap with each other, or whether a temporal interval is targetless. But there are also alternative conceptualizations on the sequencing of targets. One is gestural blending and the other is intermittent target specifications. Gestural blending is seen in articulatory phonology [12], which assumes that gestures can temporally blend with each other even for the same articulator. Gestural blending is used to explain anticipatory coarticulation as well as undershoot [12,123]. The execution of gestural blending is implemented in the task dynamic model as weighted averages of the blended gestures [12]. There is evidence, however, that the movements of any single articulatory dimension result from sequential rather overlapping execution of successive targets. This is shown for tongue body [124], velum and lips [125,126], and *f*_0_ [127]. Also, Ostry et al. [128] have demonstrated that a model based on the equilibrium point (EP) hypothesis of motor control [129] is able to generate kinematic movements that show coarticulatory overlap with non-overlapping underlying control signals. Here, a conceptual difficulty is the non-unique relations between the observed articulatory/acoustic trajectories and possible underlying control parameters, as illustrated in Figure 6.

In panel (A) of Figure 6, there are three successive target approximation movements, each largely attaining its target by the offset. These movements are strictly sequential, as indicated by the alteration of the line patterns and colors. Panel (B) also shows three target approximation movements, but the first one is shortened relative to the first movement in panel (A), resulting in undershoot, i.e., an incomplete attainment of the target. From the graph, it is clear that the undershoot is due to a premature termination of the first movement by the early onset of the second one, which *truncates* the former. But the truncation also makes the offset of the first movement appear “assimilated” to the second target, as indicated by the arrow. When the time reference (vertical line) remains unchanged from panel (A), the first movement also appears to “anticipate” the second one, although there is no true anticipation given the clearly marked movement boundary. In panel (C), instead of truncation, the final portion of movement 1 and the initial portion of movement 2 are overlapped. The overlap is implemented by inserting a new target that is the average of the first and second targets. (There are also other, more sophisticated ways of blending, e.g., averaging, suppressing, and adding [11].) This *blending* thus explicitly models an “anticipatory assimilation”. The resulting trajectory, however, is not very different from the one in panel (B) if the boundaries are ignored. (Compared to panels A and B, the second target in panel C is less fully attained. This is because the blending also shortens the target approximation movement of the second target. Thus, there is less than enough time to reach the target even though the onset of the movement is actually higher than in the other two panels. This means that despite the similarity, different assumptions about sequential arrangements do lead to slight variations in surface trajectory, making direct computational comparisons possible in future research.) Thus, truncation can generate trajectories very similar to those generated by blending, but it has the advantage of not needing to specify the amount of overlap, thus eliminating a critical degree of freedom.

Sequentiality through truncation has a number of further implications. The first is that the duration of target approximation is mostly an *extrinsic* rather than *intrinsic* property of the gesture [100], which allows it to be specified by external functions like word stress, lexical quantity, focus, boundary marking, etc. [101]. Secondly, given the frequent occurrence of truncation due to the extrinsic factors [115] and the fact that any degree of truncation is possible (even up to 100%, e.g., in syllable contraction [113]), target approximation is rarely a 0–360° full cycle. Thus, it is inappropriate to model inter-gestural alignment in terms of phase relations such as being in-phase or anti-phase [14]. This would be a further reason, in addition to those listed in Table 1, against modeling motor synchrony as physical entrainment. Finally, the massive range of possible truncations [113,115] makes it impossible to control duration through articulatory strength [130,131], because, for example, it is inconceivable that an extreme shortening of a segment or syllable up to full elimination is achieved by a maximum increase in stiffness.

#### 3.1.3. Full vs. Underspecified Targets

**Figure 7:**
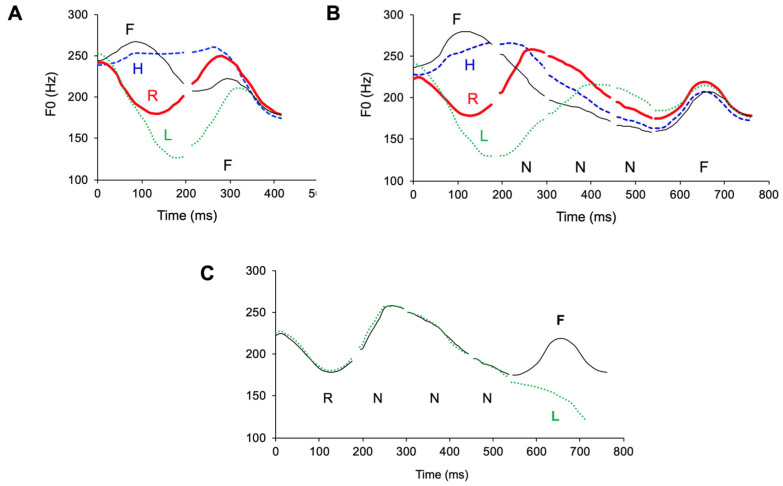
F_0_ contours of the neutral tone vs. full tones in Mandarin: (**A**) Four full tones followed by a Falling tone. (**B**) The same four tones followed by a sequence of neutral tones. (**C**) A sequence of neutral tones followed by either a Falling tone or a Low tone. Data from Chen and Xu [127]. These plots show evidence that the neutral tone has a fully specified underlying pitch target, which includes a specification for a weak articulatory strength.

Underspecification has been a popular idea in both phonology and phonetics to account for severe undershoot or lack of apparent targets [132,133,134,135]. The hypothesis is that some units do not have fully specified phonetic values, and their surface patterns come from interpolation between adjacent, fully specified units. Boyce et al. [126] have shown, however, that intervals with highly variant lip rounding and nasalization properties may still stem from specific underlying goals, as observed with minimal contrast comparisons of articulatory movements. Similar findings have been made for the neutral tone in Mandarin [112,127], which has often been considered targetless. In Figure 7A, the F_0_ contours of the Falling (F) tone in the second syllable converge quickly to a falling slope following the four full tones in the first syllable. In contrast, the neutral-tone sequences in Figure 7B do not show full convergence of F_0_ by the end of the second syllable. But by the end of the third neutral tone in Figure 7B, all the trajectories have approached a mid-level F_0_. This approximation indicates that the neutral tone has its own target, which is halfway between the Falling tone and the Low tone, as evident from Figure 7C. But the slow approximation in Figure 7B, as opposed to the quick convergence in Figure 7A, suggests a weak articulatory strength in the realization of the neutral-tone target. Note that the assumption of no underspecification further reduces DOF by eliminating the need to specify for every temporal interval whether some of the target properties are missing [12,136]. 

#### 3.1.4. Target Approximation vs. Its Preparation

**Figure 8:**
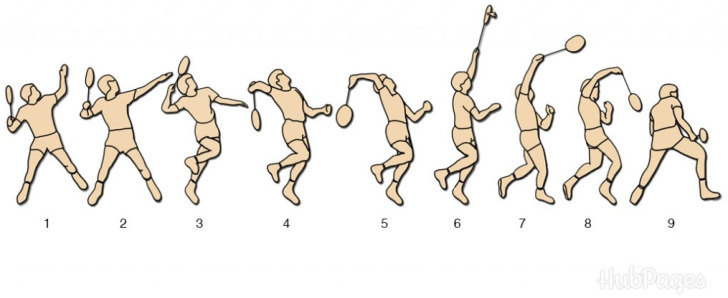
Decomposition of a badminton smash consisting of three phases. Frames 1–4 are the preparation phase, while frames 4–6 are the unidirectional approximation phase. The goal is not only to reach the position of the racket-shuttle contact, but also to achieve a high velocity at the point of contact. Frames 7–9 are the settling phase [143], (courtesy of Michael Hayes at HowTheyPlay.com).

From Figure 7C, it is also apparent that there is no anticipatory effect of the F_0_ differences due to the tone of the final syllable upon the preceding neutral tones, as shown in Figure 6. This suggests that there is no need to assume a leftward overlap of the full-tone target with the preceding target even if it is weak. But anticipatory preparation has been a popular idea for segmental articulation [68,137]. Yet the definition of preparation has been unclear. As an illustration, Figure 8 shows the decomposition of a badminton smash, a complex skilled motor action. The goal of the action is to strike the shuttlecock as hard as possible, which is achieved by a unidirectional arcing movement of the racket (frames 4–6). But before that, the racket is moving in the opposite direction *in preparation for* the main arcing movement (frames 1–4). The function of this *preparatory* movement is to maximize the travel distance for the racket during the smash, with the goal of achieving a high velocity. Similar preparatory movements have been shown for both singing and speech. For singing, a preparatory movement in the opposite direction from the target note is found to be a core property of the singing voice [138]. For speech, pre-low raising, which increases the pitch of a non-low tone before a low-pitched tone, has been reported for a number of languages [5,94,139,140,141,142].

In contrast to the preparatory movements in frames 1–4, the movements in frames 4–6 are all in the direction of making the ultimate contact, and so no part of it, including the initial portion, say frames 4–5, should be taken as preparatory activities separate from the rest of the arcing movement in the same direction. By the same token, the entire *unidirectional* movement toward a phonetic target should be considered a single-target approximation action. This distinction between preparation and target approximation will be highly relevant in the upcoming discussion of coarticulation. (It is possible, however, that even the preparatory movements in frames 3–4 of Figure 8 are part of the smash action. Whether this is the case could be determined by the timing of the movement sections. In Figure 5, e.g., the anticipatory raising movement, hence, the “preparation”, before the L and F tones seem to start from the middle of the preceding syllable. That is where the second syllable actually starts, as argued in the subsequent discussion. This possibility has been investigated in recent research, as will be discussed in Section 3.2.4.)

#### 3.1.5. Dynamic Targets and Velocity Propagation/Continuity

From Figure 5B,C, we can see that the F_0_ contours of the Falling tone after four different tones all converge to a linear falling slope, and those of the Rising tone all converge to a linear rising slope. It has also been shown that in both dynamic tones of Mandarin [95] and Cantonese [144], and diphthongs in American English [145], the final velocity of F_0_ and formants remains largely constant when the speech rate varies from normal to slow. Thus, a specific velocity is aimed at as part of the phonetic target associated with those linguistic units. Dynamic targets are actually commonplace in other motor movements. Again, from Figure 8, we can see that when the target is reached at frame 6, what is achieved is not only a particular position of the racket, but also a high-speed impact on the shuttlecock. Thus, the target of the smash is dynamic, consisting of both position and velocity specifications. Also, given a high velocity as part of the goal of a dynamic target, its achievement may have a powerful carryover effect on the following movement. In Figure 7C, for example, the final velocity of the Rising tone is so high that the F_0_ rise continues for more than half of the syllable in the following neutral tone.

#### 3.1.6. Summary of Target Approximation

There is much evidence that continuous surface movements of both articulatory and acoustic dimensions result from strictly sequential approximation of successive targets, and each approximation is executed with a specific articulatory strength. On the other hand, there are also alternative models that assume temporal overlap of auto-articulator gestures and underspecified targets, and targetless intervals. Although those models can theoretically also generate contextually variant surface trajectories, strictly sequential and fully specified targets have the advantage of assuming fewer degrees of freedom and offering a simpler basis for defining the onset and offset of articulatory gestures, which is critical for edge synchronization.

### 3.2. Edge Synchronization

As shown in Figure 2, *edge synchronization* means that (a) the onset of the syllable is the start of the target approximation for most of the syllabic components, including the initial consonant, the first vowel, the lexical tone, and the phonation register (here, phonation refers to the use of voice quality as an independent dimension to mark lexical contrasts in some languages [88,89]; it does not refer to phonation properties that accompany consonant manner of articulation.); and (b) the offset of the syllable is the end of all the remaining movements. The mechanism therefore entails full synchrony at both edges of the syllable. The synchrony is asymmetrical across the syllable, however. At the left edge, there is a synchronous onset of all the syllabic components involved, while at the right edge, there is the synchronous offset of only laryngeal components with either C or V, but not both. The benefit of synchronization is already discussed in Section 2, and the following discussion is only on the evidence and manner of its realization.

#### 3.2.1. C-V Synchronization and Coarticulation

**Figure 9:**
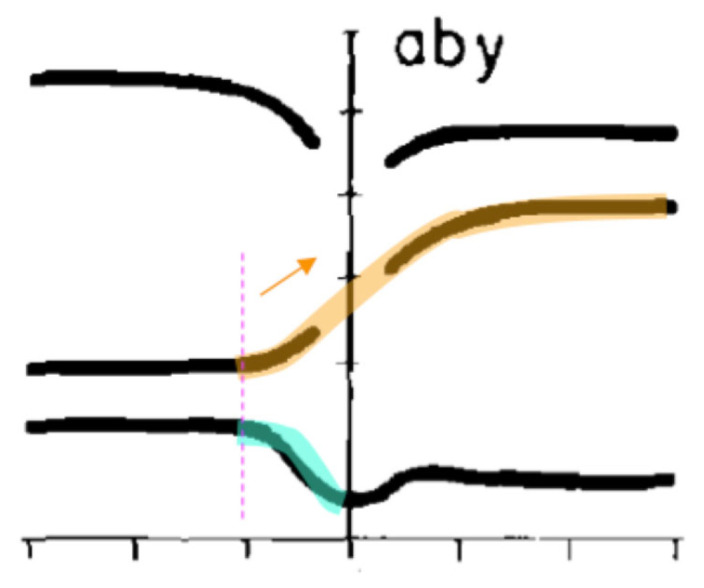
A reinterpretation of Öhman [86]. Reproduced with permission from the Acoustical Society of America, with illustrative modifications. The orange highlight tracks F2 movement toward the vowel target, while the cyan highlight tracks F1 movement toward consonant closure.

As mentioned in Section 2.4, a major objection to the notion of articulatory syllable [81] is that the scope of the vowel should extend well before the syllable onset based on the classic finding of Öhman [86]. That is, in a V_1_CV_2_ sequence, the activity of V_2_ can be seen during V_1_, as shown in Figure 9, where F2 starts to rise well before the closure of /b/. Öhman’s interpretation of this “anticipatory” activity is that “a motion toward the final vowel [V_2_] starts not much later than, or perhaps even simultaneously with, the onset of the stop-consonant gesture”. [86] (p. 165). But acoustically, the start of that activity, namely, the rise of F2, is well *inside* V_1_, which gives the impression that V_1_ is coarticulated with V_2_ [68]. Here lies, therefore, the key challenge of coarticulation: the *discrepancy* between the articulatory and acoustic onset of a phonetic unit, namely, articulation starts well ahead of acoustics. Now, the explicit definition of sequential target approximation in Section 3.1 would suggest that, in fact, *there is no discrepancy between articulation and acoustics*. This is because *any acoustic movement away from the target of a sound is by definition no longer part of that sound*. By the time F2 starts to turn upward in Figure 9, the articulation of V_1_ (/a/) is already over, and the articulation of V_2_ (/y/) has already begun, as illustrated in Figure 6B. There is therefore no evidence of anticipatory coarticulation of V_2_ with V_1_ from Öhman [86].

But true coarticulation in the sense of co-production [146] does happen, between syllable-initial consonant and the first vowel of the syllable. In Figure 9, roughly at the time when F2 makes an upward turn, F1 starts to go down toward the low valley in the /b/ closure, indicating that the articulation of /b/ also starts from there. What is not clear is whether the target approximations of V_1_ and C happen exactly at the same time, as Öhman [86] did not directly compare the timing of the articulation of C and V. And, despite the postulation of the synchronous C-V co-onset resulting from planning gestural oscillation [13], subsequent studies under articulatory phonology framework have repeatedly reported asynchronous C-V onsets [147,148,149,150,151]. This has led to the declaration that the newly accepted generalization in AP is that the vowel gesture starts somewhere after the onset of the consonant closure gesture but before the release gesture [152]. But as pointed out in Liu et al. [153], the CV asynchrony found in these studies is due to a flawed method of determining gestural onsets based on velocity profiles of individual gestures themselves, which is susceptible to confounding from adjacent and concurrent gestures and variable intrinsic stiffness of different gestures.

**Figure 10:**
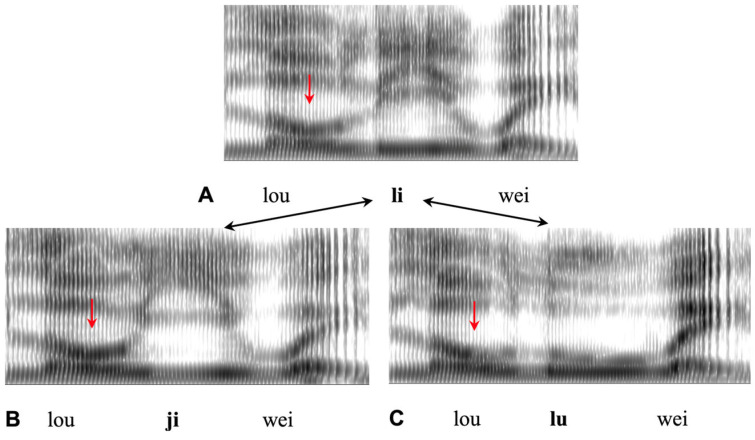
Example spectrograms of a C_1_V_1_#C_2_V_2_ minimal triplet from [155]. The three-syllable sequence at the top serves as a control for both sequences at the bottom: /li/ in (**A**) contrasts with /ji/ in (**B**) for C_2_: /l/ vs. /j/, and with /lu/ in (**C**) for V_2_: /i/ vs. /u/. The red arrows point to the common divergent points for both C and V.

Clearer evidence is found in a series of studies based on a minimal triplet (or double minimal pair) method proposed in [154], which uses the gestural divergent point of C and V minimal pairs as indicators of their respective onsets, and determines CV synchrony by comparing the timing of these onsets. In Xu and Gao [155], for example, the stimuli were minimal triplets of syllable sequences in Mandarin in the form of C_1_V_1_#C_2_V_2_, as shown in Figure 10. Each triplet consists of two minimal pairs. The first contrasts the two consonants in C_2_: [j] vs. [l], while the second contrasts the two vowels in V_2_: [i] vs [u]. In the first minimal pair, the divergent point of the F2 trajectories indicates the onset of C_2_, because that is where the two consonants start the approximation of their respective places of articulation. In the second minimal pair, the divergent point of F2 indicates the onset of V_2_, because it is where the two vowels start the approximation of their respective vocal tract shapes. The two consonants are both sonorants with incomplete closure of the oral cavity, so that formant movements during the consonantal constrictions can be traced. In addition, all the words have a Rising tone on both syllables, which allows the two resulting F_0_ peaks to serve as time references for the onset and offset of the second syllable [156]. 

**Figure 11:**
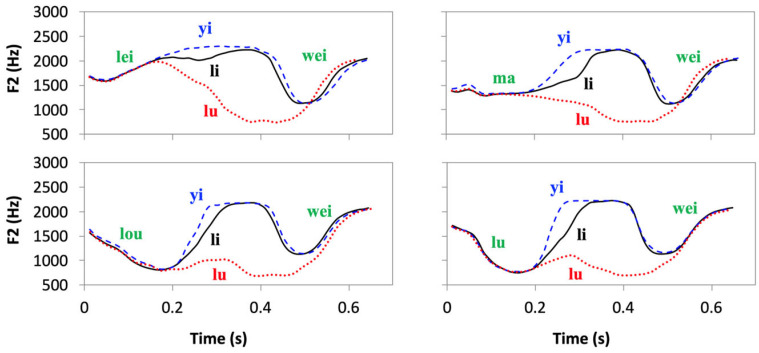
Mean F2 trajectories of the middle three syllables in the Mandarin sentence frame “bi __ wei shan” [more hypocritical than __ ], where the keyword is a disyllabic personal name. The three trajectories in each plot differ either in the initial consonant or the vowel, and the four plots differ only in the first syllable. The time of all trajectories is relative to the voice onset of /bi/ in the carrier. Both F2 and time are averaged across 8 repetitions by 3 male speakers. (Data from [155]).

Figure 11 shows the mean F2 trajectories of four of the triplets in [155] produced by three male speakers. In each plot, the solid and dashed lines differ in the initial consonants: [l] vs. [j], and the point at which the two trajectories start to diverge indicates the onset of both consonants. The solid and dotted lines, on the other hand, differ in the vowels of the second syllable: [i] vs. [u], and the point at which the two trajectories start to diverge indicates the onset of both vowels. Strikingly, in each case, the vowel divergent point occurs at about the same time as the consonant divergent point. This common divergent point, as indicated by the vertical arrows in Figure 10, is well ahead of the onset of the [l] closure. This finding is recently supported by a more systematic study on Mandarin, with parallel articulatory (EMMA) and acoustic data and Bayesian statistics [153]. In addition, also using the minimal triplet paradigm, Liu and Xu [157] showed that in CCV syllables in British English, the vowel gesture starts in synchrony with the very first consonant, just like what is described for Russian [81]. (Note that this finding contradicts the C-center model, which aligns the vowel onset to the center [158]. But it is important to note that the C-center effect refers to the consistent duration of the conventional vowels in syllables with consonant clusters of various lengths. As such it does not directly assess the actual alignment of vowels with the consonant clusters.) There is therefore rather clear evidence by now that consonant and vowel target approximations start at the same time at the onset of the syllable.

#### 3.2.2. Coarticulation Resistance

The coproduction of C and V at the syllable onset means that they would interfere with each other’s articulation because they often have conflicting goals. This would result in variations in their acoustic output. Given coproduction as the mechanism of coarticulation, however, there has to be a solution to the articulatory conflict between the coproduced phonetic components. It is known that some segments show better ability to resist coarticulatory variation [159,160,161]. A major source of such *coarticulation resistance* is the amount of constraint that a consonant or vowel places on the tongue body [160,161]. Those with intrinsically stronger tongue body constraints show greater resistance to coarticulatory influence than those with weaker constraints.

What can be first recognized is that the severity of the conflict would depend on the number of articulators shared by the co-produced sounds. The least conflict occurs between well-separated articulators, e.g., the larynx and the oral articulators, as will be discussed in Section 3.2.4. The most severe conflict would occur when virtually all the articulators receive clashing demands. This would happen between glides like /i/ or /w/ and the following vowel. As semivowels, their articulatory targets specify the shape of the entire vocal tract, just like a vowel. The glide and vowel targets, therefore, have to be sequentially approximated, as can be seen in Figure 10B for /w/ between /i/ and /ei/. If some of the articulators are shared while others are free to serve either of the two sounds involved, an obvious solution is for the shared articulators to sequentially approach different targets, while allowing the rest of the articulators to simultaneously approach their respective targets. In /ba/, for example, the shared articulator, the lips, can first make the bilabial closure and then open up for the vowel. At the same time, all the lingual articulators, with no positional requirement from /b/, would be free to start approaching their /a/-specific targets from the syllable onset.

The trickiest situation is when two segments share the same *primary articulator*, as in the case of velar consonants like /ga/ and /ka/. Because the tongue body needs to contact the velum, there is a direct conflict with the coproduced vowels, which also have clear specifications for the tongue body. How is it possible, then, for sequential target approximation to resolve the articulatory conflict? Our hypothesis, as already hinted in Section 3.1.2, is dimension-specific sequential target approximation (DSSTA); that is, sequential target approximation can be specific to individual dimensions of an articulator rather than always over the whole articulator. For the velar consonants, as the tongue dorsum needs to be raised to make the velar contact, the vertical position of the tongue dorsum therefore has to first approach the consonant target before turning to the vowel target. The precise horizontal position of the tongue body, in contrast, is probably less critical for the consonant. Therefore, the horizontal position of the tongue dorsum can start to move toward the vowel target right from the syllable onset. As a result, the point of contact between the tongue body and the palate for /k/ would naturally vary gradiently with the coproduced vowel: more advanced for front vowels, and more retracted for back vowels [162]. Conceptually, therefore, *dimension-specific* sequential target approximation not only resolves the problem of coarticulation resistance but also explains how CV co-onset is articulatorily implemented in general. 

Two recent modeling studies have put DSSTA to the test. In [163], an articulatory synthesizer [164] in which the dynamics of all articulators are controlled by a target approximation model, was trained with acoustic signals of CVC syllables to learn articulatory targets of consonants and vowels. During learning, the training algorithm allowed tongue dorsum height to be controlled by the velar stop up until the moment of tightest closure, and the tongue dorsum frontness was controlled by the vowel from syllable onset to vowel offset. Not only was the variable velum contact location successfully learned, but also the gV syllables synthesized with articulatory parameters learned this way were highly intelligible, with mean recognition rates of 78%, 100%, and 83% for *get*, *god*, and *good*, respectively. In [121], the application of DSSTA was found to be effective in the control of not only tongue dorsum in velar stops, but also other articulators in bilabial, alveolar, as well as velar stops. 

#### 3.2.3. Locus and Locus Equations

**Figure 12:**
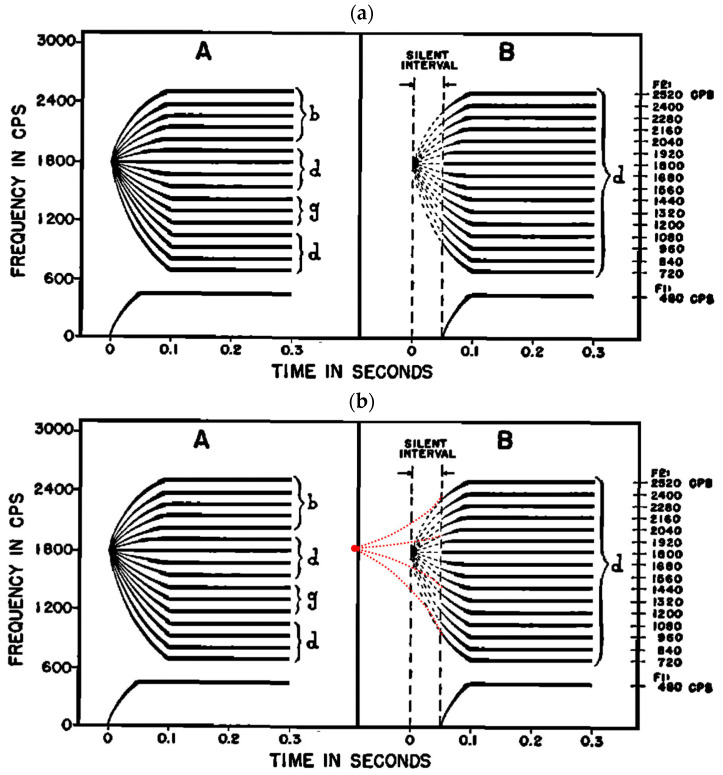
(**a**) An illustration of the locus phenomenon [166]. Reproduced with permission from the Acoustical Society of America). The curves are F1 (the curves near the bottom in both plots), and F2 hand-painted for the pattern playback speech synthesizer [168]. b, d, and g mark how listeners identified the consonants. (**b**) A reinterpretation of [166] with added virtual F2 traces in red. In A, F2 is fully continuous; in B, the initial portion of F2 is silenced.

Coarticulation resistance is also closely related to two other classical phenomena, namely, locus and locus equations. Locus is a phenomenon observed soon after the classical discovery that F2 transitions carry perceptual cues for consonant place of articulation [165]. It was found that many of the transitions point back in time to a *locus* such that as long as the first part of the transition is silent, the same consonant is heard [166,167], as shown in B in Figure 12a for /d/. The presence of the entire transition would, in contrast, result in hearing different consonants depending on the following vowel (A in Figure 12a). As reasoned by Delattre et al. [166] (p. 772), the locus phenomenon indicates that “no appreciable sound is produced until at least part of the articulatory movement has been completed”. But it is left unexplained *why* the early part of the transition movement has to be silent.

Closely related to the locus phenomenon are locus equations [88,169]. Locus equations refer to the finding that the onset of vowel F2 transition after a given stop consonant (i.e., the equivalent of the second vertical dash in Figure 12b(B)), when plotted over F2 at the “center” of the vowel (equivalent of the plateau in Figure 12b(B)) across different vowels, shows a strong linear relation. Fowler [170] and Iskarous et al. [171] have linked the linearity in the locus equations to coarticulation resistance, arguing that it demonstrates the invariance in coarticulation resistance across different vowels. Lindblom and Sussman [169] have further linked the locus equation back to the classical locus phenomenon, proposing that the critical articulation of a stop consonant is the target: lips for /b/, tongue blade for /d/, and tongue body for /g/, but the rest of the articulators have no specified target and so are allowed to be coarticulated with the vowel. This account comes very close to the coarticulation resistance account by Fowler and colleagues. Common to both accounts, however, is that they are concerned only with the articulatory or formant movement from the *voice onset* to the center of the vowel. Lindblom and Sussman ([169] p. 17) explicitly assumed that “the movements of the articulators in a CV syllable start from their positions at stop closure”.

Based on the discussion of edge synchronization thus far, the movement toward the vowel in a CV syllable starts *neither* from the voice onset after the consonant release, *nor* from inside the closure, but from the onset of the final formant transition in the preceding syllable, as illustrated by the dotted curves in (B) of Figure 12b. Even if the syllable is utterance initial, as in the case of Delattre et al. [166], vowel target approximation also would have begun *before* the consonant closure, i.e., at the time when the consonant closure *starts* to form. Hence, the onset of the visible formant transition is well *after* the onset of vowel articulation. This perspective has two implications. Firstly, the linearity of the locus equations is largely due to a part-whole correlation [172,173,174], since the two F2 measurements are taken from two locations along the same unidirectional movement from the consonant to the vowel: voice onset, which is virtually a halfway point of the C-V transition, and *center* of the vowel, which is the end of the vowel target approximation. Secondly, because the vowel is coproduced with the consonant at syllable onset, and coarticulation resistance is the result of coproduction (depicted in (B) in Figure 12b as the warping of the dotted curves) whose severity depends on how much the consonant articulation conflicts with that of the vowel, to the extent resolvable by DSSTA, the slope of the locus equations would naturally reflect the amount of coarticulation resistance. DSSTA, therefore, has offered an ultimate solution to the inevitable conflict between consonants and vowels when they have to be coproduced, as per demand of CV synchronization.

#### 3.2.4. Synchronization of Laryngeal and Supralaryngeal Articulations

As early as 1984, Ohala and Kawasaki suggested that “the division of sound sequences into syllables” is “for the sake of synchronizing the segmental and suprasegmental articulations” [175] (p. 123). This proposal foreshadowed a series of later findings in both tone and non-tone languages that generated five lines of evidence for the full synchrony of tone and syllables. The first line of evidence is that the *start of tonal movement is aligned to syllable onset*. The clearest cases are from tone languages, for which it is possible to directly observe where different tones start to move toward their respective targets [5,93,94,95]. In Figure 5, for example, in each plot, the F_0_ contours of the four tones start to diverge roughly from the onset of the second syllable toward their respective targets. A similar consistent start of F_0_ movement toward tonal targets at syllable onset has also been reported for Cantonese [140,144] and Shanghai Chinese [176]. For non-tone languages, there have been many findings of systematic alignment of F_0_ turning point to syllable onset, e.g., Dutch [177,178], Spanish [179], Greek [180], English [181,182], Italian [183], Portuguese [184], German [185], Arabic [186], and Persian [187].

The second line of evidence is that *tonal target approximation starts from syllable onset even if the initial consonant is voiceless*. As found in Mandarin [188], Cantonese [189], and English [190], when F_0_ contours are time-normalized with respect to the syllable, they parallel each other closely whether the onset consonant is sonorant as in [ma] or voiceless as in [ta], [t^h^a], or [ʂa]. Thus, the approximation of the underlying tonal target starts not from the voice onset, but from the beginning of the syllable, regardless of whether the vocal folds are vibrating during the initial consonant. 

The third line of evidence is that *tonal target approximation ends at syllable offset even if there is a coda consonant.* As found in [93], in disyllabic words with or without a nasal coda in the first syllable, the final part of tonal target approximation is executed through the coda nasal as if it is part of the first syllable. This indicates that the entire syllable is the domain of tonal target approximation whether or not it has a nasal coda, as long as the next syllable does not start with an approximant or a vowel.

The fourth line of evidence is that synchronization of laryngeal and supralaryngeal articulation may benefit vocal learning by eliminating temporal degrees of freedom [191]. As simulated with the qTA model, the learning of tones from raw *f*_0_ contours in real-speech data yields both faster and more efficient tone learning when tonal targets are assumed to be fully synchronized with the syllable than when the tone–syllable alignment is assumed to be flexible and has to be learned.

Finally, if C and V both start their target approximation earlier than the conventional syllable boundary as has been argued, then so should lexical tones. Initial evidence for the early tone onset can be seen in Figure 5 where the H tone in syllable 3 is followed by the L tone. F_0_ starts to drop toward the low target not at the conventional syllable boundary where the vertical line is drawn, but well before it (but also see discussion in Section 3.1.4). More solid evidence has been shown in [192], which applied the method developed in [153]. The results show that tone and vowel onsets are fully synchronized, which in turn indicates full synchrony of tone and syllable. Most surprisingly, with the newly established tone onset, the ‘anticipatory raising’ effect of tone is found to occur *within* rather than *before* the articulatory syllable. What this suggests is that the preparatory move discussed in Section 3.1.4 may be of two kinds, as can be seen in Figure 8. A slower one is from frame 1 to frame 3, which involves a backward extension of the arm and shoulder, and a fast one between frames 3 and 4, which involves mainly a backward flexion of the wrist. The anticipatory raising effect of the Low tone seems to be more like the fast preparatory wrist flexion, given its briefness. The finding that it happens within the syllable is astounding, and may have implications for many nonspeech motor movements as well.

#### 3.2.5. Vowel Harmony, an Unresolved Issue

Vowel harmony is the phenomenon that in some languages, within a relatively large temporal domain such as a word or phrase, there is a tendency for vowels to share a particular property along a phonetic dimension, such as tongue height, or tongue frontness [87,193]. Such long-distance assimilation can be either left to right or right to left. Related to the right-to-left harmony is the long-distance anticipatory vowel-to-vowel assimilation across more than one syllable reported for English, which is referred to as a form of coarticulation [194,195]. Both kinds of long-distance assimilation seem incompatible with either target approximation or TD/AP, as it would mean that the approximation of a single vowel target can occur across multiple target approximation movements of the intervening consonants and vowels. The target approximation model, however, allows a distinction between target assignment and target approximation [196]. That is, it is possible for the target of a phone, be it segmental or suprasegmental, to be modified, or *reassigned*, before the start of its articulatory execution. The target reassignment, if extensive enough to be heard as a different vowel, could have originated historically from surface assimilation [197], due to listeners’ misperception [175,198]. Or it could be a small target readjustment in anticipation of an upcoming vowel [193] or consonant [199,200]. The separation of target assignment and target approximation as different processes means that only the latter involves genuine coarticulation, which is universal, while the former is due to a separate mechanism that is language specific, as has been demonstrated in a preliminary study of French [201].

#### 3.2.6. Summary and Implications of Edge Synchronization

Multiple lines of evidence have been presented that consonants, vowels, and tones are likely synchronized by their onset at the beginning of a syllable. There is also a synchronization tendency at the offset of the syllable, although the evidence is only in terms of tone–syllable alignment. More discussion of syllable offset will occur in light of tactile anchoring in the next Section. 

**Figure 13:**
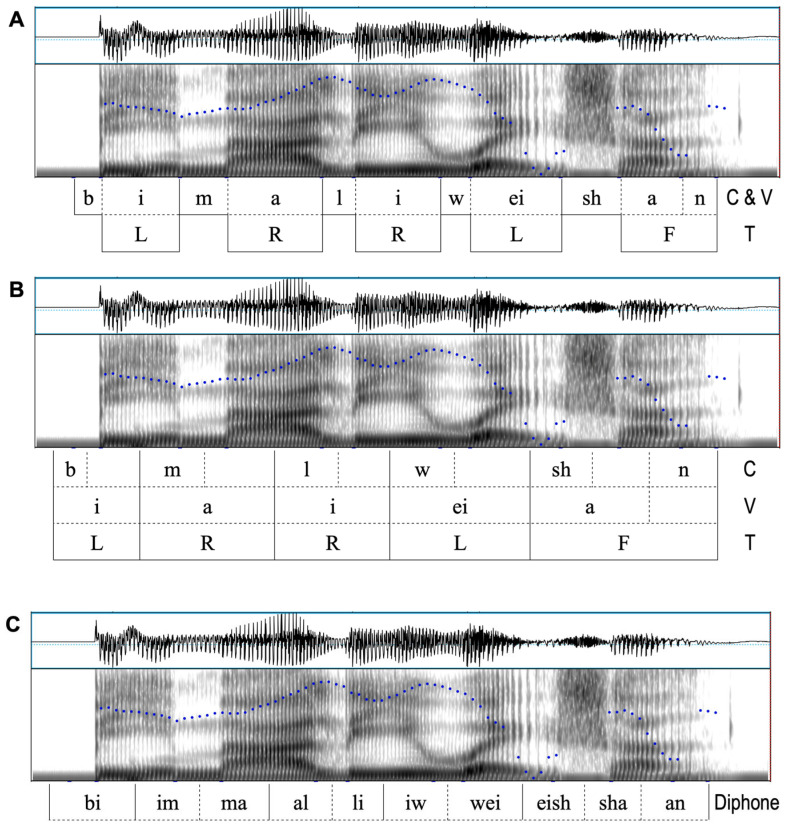
Spectrogram of the Mandarin phrase “比麻黎伪善” /bǐ má lí wěi shàn/ [more hypocritical than Ma Li] [155], with broad phonetic transcriptions and pitch tracts (in blue). In both panels, C, V, and T stand for consonant, vowel, and tone, respectively. In (**A**), the segmentation is conventional [63,64]. The segmentation of /w/ is based on [65]. In (**B**) the segmentation is based on the synchronization hypothesis. In (**C**) the segmentation is based on the diphone principle [202,203].

A major impact of the edge synchronization hypothesis is an overhaul to the way we conceptually segment speech, as illustrated in Figure 13. Unlike the conventional segmentation in Figure 13A, in Figure 13B, the onset of each segment is at a time when the spectrogram *starts to move toward* its prototypical configuration. The first /a/, for example, is in the middle of the conventional /i/ where F2 starts to drop, and the second /i/ is in the middle of the conventional /a/ where F2 starts to rise. Thus, the onset of a vowel is fully aligned to the onset of the initial consonant, which is also shifted leftward: to where an oral closure just starts to form, as indicated by the downward turn of F1. As explained earlier, the large leftward shift of a vowel onset (by about 100 ms) is a conceptual change that can explain away much of the anticipatory V-to-V coarticulation. The new *offset* of a segment is where the spectral pattern has maximally approached its canonical configuration and started to move away from it. For /i/, it is at the peak of F2 and F3, for /a/, it is at the peak of F1 and the valley of F2, and for /w/, it is at the valley of F2. For the obstruent consonants, the offset is not at the end of its prototypical spectral pattern (e.g., closure gap in /b/, nasal or lateral formants in /m/ and /l/, and the frication in /ʂ/), but in the middle of these intervals. Furthermore, a coda consonant, e.g., /n/ in /shan/, is aligned *after* the nuclear vowel, *without overlap*. The reason for the lack of VC overlap will be discussed in Section 3.3.2. Finally, tones are fully synchronized with the entire CV or CVC syllable, as shown in the bottom tier.

Just as importantly, Figure 13B makes it clear that the coproduction of consonants and vowels is in fact *acoustically transparent* rather than hidden. For example, the rise of F2 and F3 toward the high extremes of /i/ in /li/ from the middle of the conventional /a/ is clearly visible. Also apparent is the start of the F1 drop toward the low extreme in /l/ from the middle of the conventional /a/, which marks the onset of oral closure for both /l/ and /i/. Currently, these visible patterns are given names like transitions, anticipatory movements, etc. The new segmentation treats them, instead, as belonging to the main bodies of the segments.

The representation of the true segmental intervals by the new segmentation shown in Figure 13B is in fact foreshadowed by the concept of diphone in speech technology [203]. A diphone is defined as an acoustic chunk consisting of two adjacent halves of a pair of conventionally segmented phones [202,203]. As illustrated in Figure 13C, each diphone extends from the middle of one conventional phone to the middle of the next. Intriguingly, the diphone boundaries in Figure 13C match well with those of the new segmental intervals in Figure 13B. This means that a diphone actually represents a single phone rather than two phones in many cases, especially in the case of consonants. For example, the diphone [im], [al], [iw], and [eish] in Figure 13C actually represent the full scope of [m], [l], [w], and [sh], respectively. But the diphone representation of vowels is incomplete, compared to the new segmentation scheme, because it misses the initial portion of the vowel. For example, the vowel interval in [ma] in Figure 13B spans across two diphones in Figure 13C: [im] and [ma], and the vowel interval in [li] spans across [al] and [li]. Missing the initial portion of the vowel in the diphone segmentation therefore is likely a major reason why so many contextual features are needed in training a diphone synthesis or recognition system.

### 3.3. Tactile Anchoring

Tactile anchoring is about how synchronization is achieved in speech production, and it may hold the key to understanding some of the structural details about the syllable. The hypothesis is that the accuracy of edge synchronization rests on sensory feedback, and that tactile sensations generated during articulation likely provide the most useful feedback information. It follows that the points of synchronization are at the syllable edges rather than in the center of the syllable. Most previous theories of the syllable regard the center, where sonority is the highest, as the core of the syllable (see detailed review in [204]). Tactile anchoring predicts, in contrast, that the center of the syllable, where contact sensation is likely weak, would be the least reliable anchor.

#### 3.3.1. Why Is Tactile Anchoring Needed?

One of the earliest clues comes from the finding that, just like bimanual synchronization [24], concurrent leg swinging by two people sitting next to each other also shows stable phase relations only at 0° and 180°, and 0° is the only stable relation at high speed [27]. But this holds only when the participating subjects can see each other’s movements. This perceptual nature of motor synchrony is further demonstrated by Mechsner et al. [26], which shows that the propensity for, as well as the ability to achieve bimanual synchrony are *perceptual* in nature. They demonstrate that naïve subjects are able to perform bimanual oscillations in a 4:3 frequency ratio, which are virtually impossible to maintain based purely on body-oriented strategies, as long as they can *see* a 1:1 frequency ratio converted from their actions by a mechanical device. Besides visual perception, tactile [205,206,207,208] and proprioceptive [209,210,211,212,213] information has also been shown to help stabilize in-phase coordination in bimanual tasks. Thus, the perceptual guidance needed for motor synchrony includes any sensory feedback, and the importance of each perceptual channel is a function of the *clarity* of the feedback information it provides to the central control system. 

For speech, to ensure synchrony in syllable articulation, visual feedback is unlikely to be useful, as speakers cannot see their own articulators. Auditory feedback is available all the time and is likely very useful [214], but it may not be the most critical, as people who become deaf post-lingually are often able to speak intelligibly for decades [215,216]. Also abundantly available is proprioceptive feedback during speech, but the information it provides is likely spread evenly over time; thus, it may be useful, but it is not the most critical. The sensory information that probably fluctuates the most with the opening and closing of the vocal tract is from tactile feedback, especially from the articulators that are rich in tactile receptors, such as tongue tip, tongue blade, and the lips [217,218], whose sensitivity “rivals or exceeds that of the fingertip” [219]. This points to consonant closures as the most likely sites of tactile anchoring, because they are brief, easily palpable, and exact in time.

#### 3.3.2. Evidence for Tactile Anchoring in Speech

Tactile anchoring is the most speculative component of the new syllable theory as there is only indirect evidence so far. The first is that blocking tactile feedback in the oral cavity through topical anesthesia not only lowers intelligibility, but also reduces speech rate [220], presumably because more time is needed to ensure tactile anchoring. The second is that in consonants that involve multiple articulatory components, the gestural components with a tighter oral contact tend to be aligned closer to syllable edges. In English, for example, the apical gesture in /l/ reaches its extreme near the syllable margin, whereas its dorsal component reaches the extreme closer to the nuclear vowel, whether /l/ is a coda (hence, the dark variant) or an onset (hence, the light variant) [221]. This means that gestures that generate clearer tactile feedback are preferred at syllable edges over those that provide less clear tactile information. That is, the apical gesture of /l/ involves a tongue tip contact with the alveolar ridge; hence, the rich tactile sensation at the tongue tip provides much more sensory feedback than the more vowel-like tongue body gesture [218]. A similar finding is that in /w/, the labial gesture is also more peripheral than the tongue body gesture [222]. Not only does the labial gesture of /w/ involve more skin contact than the tongue body gesture, but also the lips have a rich sensory representation [218].

The second phenomenon is the well-known onset-coda asymmetry; i.e., CV syllables are much more common than VC and CVC syllables, both within and across languages [47,62,69,75,175,223,224]. Even if they are already present in a language, coda consonants are more vulnerable than onset consonants, as they are subject to reduction, deletion, and resyllabification [61,79,225,226]. The vulnerability of the coda means that it is not as reliable as the onset for providing tactile feedback. As for why the onset/coda asymmetry is there in the first place, there are a number of possible reasons. First, syllable onset is where the greatest number of syllabic components can be synchronized, including consonant, vowel, and tone, as mentioned before. In contrast, syllable offset can end with either a vowel or a coda consonant, but not both. This is because the closure of a coda consonant is in direct conflict with the opening movement of the preceding vowel. This differs from the syllable onset, where it is tolerable for a vowel to be briefly interrupted by the closing movement of the initial consonant. The sequential articulation of codas is likely one of the major reasons for their vulnerability to reduction and deletion [114]. As a syllable shortens when the speech rate increases, there is less and less time left to allow sequential execution of multiple segments within the syllable [113]. This vulnerability means that syllable onset is the only temporal location for generating reliable tactile input. Furthermore, because target approximation is frequently incomplete [115], and different syllabic components may have different degrees of incomplete approximation, synchronizing their offsets is hard. Most importantly, syllable offset is also the onset of the next syllable, which already provides a synchronization point. So, there is no need for a coda to perform synchronization except at the end of an utterance.

The onset-coda asymmetry is also reflected in the resyllabification phenomenon, whereby a coda consonant goes through a change that makes it sound like the onset of the next syllable. This may happen within a word, e.g., *ending*, *producing*, which becomes *en–ding*, *pro–du–cing*, or across words, e.g., *let us*, *thin air*, which become *le–tus*, *thi–nair*. In language teaching, such resyllabification (often referred to as linking) is considered a good marker of fluency for languages like English, as non-native speakers often fail to do it [227,228]. There is doubt, however, as to whether resyllabification actually occurs, especially across word boundaries [35]. One of the reasons is that studies of resyllabification have generated diverse findings based on the researcher’s own intuition, native listener’s judgment [77,78,79,229,230], phonotactic analysis [70,71,76], or language-specific phonetic properties [35,231]. 

In Liu and Xu [232], we used a more objective method to determine syllable affiliation of intervocalic nasals at word boundaries in Southern British English. We used both singleton and cluster consonants spoken at a slow speech rate as benchmarks for canonical onset and coda consonants, and then used deep learning and dynamic time warping (DTW) to determine if some of the codas at a normal speech rate are classified as onset consonants. The majority of codas at a normal rate were indeed identified as onsets by the slow-speech-trained classifier, and the resyllabified codas were acoustically similar to their canonical onset counterparts. Also, the resyllabified coda consonants contained the same amount of information for the vowel of the second syllable as the canonical onset consonants, indicating that the resyllabified consonants were indeed coarticulated with the following vowel.

The propensity for resyllabification is further seen in a phenomenon first observed by Stetson [56]. He found that a CVC sequence such as *pup*, *pup*, *pup*…, when spoken at an increasing speech rate, changes abruptly at some point to a CV sequence: *pu*, *pu*, *pu*…. Kelso et al. [28], in a more formal experiment, show that a VC sequence like *ip*, *ip*, *ip*… also changes abruptly to *pi*, *pi*, *pi* … when the speaking rate is increased to about four syllables/s. A similar finding was reached by de Jong [233]. An abrupt shift at four syllables/s is striking, since normal speech rate is much faster, at about 5–7 syllables/s [6,234]. This means that resyllabification is virtually inevitable in normal speech, as is found in [232], unless codas are deleted when the following syllable starts with a vowel, as is the case in Mandarin [226].

#### 3.3.3. Summary of Tactile Anchoring

The need for tactile anchoring is evident from the finding that the quality of bimanual synchrony of cyclic movements is contingent on the quality of perceptual guidance during the execution of a synchronization task ([26] and many others cited above). Assuming that motor synchrony is the essence of the syllable as currently hypothesized, its accuracy would require clear feedback guidance. Of all the sensory channels available during speech production, the intermittently fluctuating tactile feedback from consonants provides the most precise feedback. Given the vulnerability of codas, the only temporal location for tactile feedback is syllable onset. This hypothesis is supported by the fact that in sonorant consonants /l/ and /w/, the tongue tip and labial gestures that generate rich tactile information are realized near the syllable edges, while the tongue body gestures are realized toward the center of the syllable [221,222], and by the onset-coda asymmetry that strongly favors CV over VC or CVC. Further support in terms of the onset-coda asymmetry comes from the strong tendency for resyllabification of coda consonants to the onset of the next syllable [28,56,232,233]. One thing that is unclear, however, is how frequently tactical anchoring is needed. The many cases of vowel hiatus across syllable boundaries suggest that it is not mandatory for every syllable, but the strong propensity for resyllabification in languages like English and Dutch suggests that it is likely to be as frequent as possible. But this is still an open question for future research.

### 3.4. What Is New Compared to Previous Theories

As mentioned in Section 2, most of the well-known theories of the syllable, including the maximal onset principle [71,72,73], the sonority theories [71,235,236,237], the phonotactic theories [46,47,70], are about syllabification; i.e., how to divide continuous texts into separate syllables. None of them is particularly concerned with how syllables are articulated—the main focus of the current hypothesis. Theories that do consider the articulation of syllables, e.g., the chest pulse theory [56], the C/D model [57], and the frame/content theory [58], are not concerned about the DOF problem. Even the coupled-oscillator model [13] and the time structure model of the syllable [114], which both proposed CV synchronization, did not discuss the problem of degrees of freedom. Thus, the current hypothesis is the only theory, to our knowledge, that posits the syllable as a synchronization mechanism for solving the DOF problem to make human speech possible.

The synchronization principle may nevertheless be relevant to the issue of syllabification, however, as it suggests a novel way of identifying syllable boundaries that are based on articulatory timing rather than phonotactics. For example, it is shown that the coda consonant in the first syllable in a CVC#VC sequence in English is resyllabified into the following VC syllable based on evidence consistent with the synchronization principle [232]. And it is also shown that in an English CCV syllable, V is articulatorily aligned with the first C [157], which suggests the possibility that even in a C_1_V_1_C_2_# C_3_V_2_C_4_ sequence, the coda C_2_ of the first syllable may also become aligned with V_2_, thus forming a C_2_C_3_ cluster that is likely non-canonical in a language. But this has yet to be empirically tested.

The synchronization hypothesis also differs from other models of the syllable in that it is able to address issues beyond syllabification. Through the proposal of the DSSTA mechanism that resolves the conflict between consonant and vocalic articulation, coherent explanations can be offered for phenomena like coarticulation resistance, locus, and locus equation. And the inclusion of laryngeal gestures in the synchronization model enables the hypothesis to be connected to research on tone and intonation, thus fulfilling the prophecy of Ohala and Kawasaki [175].

## 4. Neural Prerequisites for Syllable Articulation

The discussion so far has presented arguments from the perspective of motor control regarding why the syllable is likely a synchronization mechanism for reducing degrees of freedom to make speech possible. No neural evidence has been presented, however, because there are no neural findings, to our knowledge, that would directly implicate a synchronization mechanism. More importantly, we are of the view that speech-related neural activities serve the purpose of making speech communication possible, rather than the latter being the byproduct of the former. The three core mechanisms of the synchronization hypothesis, nevertheless, may suggest specific neural substrates that are needed for the articulation of the syllable. 

*Target approximation*, as discussed in Section 3 (cf., in particular, the graphic illustration in Figure 4 and Figure 6), implies that the neural commands sent to the articulatory muscles are in the form of underlying targets rather than either surface displacement or velocity [238,239] trajectories. Only in this way can the contextual variability in surface acoustic trajectories due to physical laws, mainly inertia, be articulatorily generated. Target approximation points out a clear forward relation from articulation to acoustics, which can be learned through *analysis by synthesis* [240]. In this process, the articulatory system repeatedly generates surface trajectories until a best fit is found, and the articulatory maneuver that can generate the best fit is stored as the learned target, as has been computationally simulated [111,119,163]. Analysis by synthesis may require that continuous acoustic signals remain available during learning to serve as auditory templates for imitative learning [241,242]. A recent set of computational simulations of speech acquisition showed that, surprisingly, the most effective way of learning the underlying target of consonants, vowels in English [117,120,121] and tones in Mandarin [243], is under the guidance of trained speech recognizers that simulate speech perception. This suggests that, while articulatory targets may indeed require separate neural representations, the auditory representation needed for guiding the acquisition of the articulatory targets is likely only implicit in the perceptual neural network rather than being localized in the brain.

Note that these latest findings have shown evidence that vocal learning is unlikely driven by online feedback control as is assumed in some of the most influential neural models, such as DIVA and HSFC [244]. Rather, vocal learning is more likely a process of prolonged trial-and-error target discovery, guided by phonological perception, with little or no immediate sensory feedback correction. The often-observed auditory feedback correction (due to the ease of laboratory elicitation) is likely an entirely separate neural process with the sole function of calibrating the articulatory-to-acoustic relation, just like the tuning of a musical instrument. Target approximation, in contrast, is hard to be immediately corrected not only because of the slow reaction time [245], but also because target undershoot happens all the time as a function of syllable duration [115]. The motor control of target approximation, therefore, likely relies entirely on a feedforward neural mechanism [238].

For *edge synchronization*, two critical neural mechanisms need to be in place. Firstly, there should be centrally generated signals for initiating each syllable. These signals, probably in the form of neural pulses, cannot be periodic, however, because their intervals need to frequently change with linguistic and paralinguistic factors such as stress [246], position in word and phrase [247,248], prosodic focus [94,233], and speaking style [249]. This view is consistent with findings of neural-based research on music and language [250]. Secondly, there should be neural mechanisms to coactivate all the involved articulators without significant time delay or discrepancy. One way to achieve this is to bring the neural control areas close to each other in the brain to ensure rapid communication. This may indeed have happened during the evolution of the human brain. Belyk [251] (p. 180) suggests that an evolutionary reorganization has brought expiration, phonation, and articulation into proximity in the brain, creating a *small-world architecture* [252,253] that would function efficiently. It would be interesting to examine in future research whether and how exactly this small-world architecture enables the synchronization of laryngeal, supralaryngeal, consonantal, and vocalic articulations.

The likely neural prerequisite for edge synchronization has been suggested by a recent neural theory of the evolutionary basis of beat-based dancing behavior shared by only humans and parrots [254]. The theory notes the human–parrot similarities in both movement to music and the neurobiology of advanced vocal learning, and suggests that “gene regulation changes associated with the evolution of a dorsal laryngeal pitch control pathway in ancestral humans fortuitously strengthened auditory–parietal cortical connections that support beat-based rhythmic processing” (p. 1). Hickok, in a commentary on Patel’s theory [255], suggests further that the emergence of the ability to synchronize to beats is likely a byproduct of the ability of speech to coordinate the timing of laryngeal pitch control and the supralaryngeal movements [256], which is exactly like what is proposed in the synchronization model of the syllable, short of only CV synchrony (echoing also the Ohala and Kawasaki prophecy [175]). Further work is therefore needed to reveal the neural mechanisms that underlie edge synchronization. One likely avenue is through technologies developed for treating neural disorders like epilepsy, including ECoG and SEEG [257,258], which may allow the observation of neural activities at sufficiently high time resolution corresponding to CVT synchrony.

For *tactile anchoring*, as discussed in Section 3.3, the precision of synchronization depends on the quality of sensorimotor feedback [26]. There should therefore be sensorimotor pathways that enable effective feedback control. The critical role of timing control has been recently demonstrated in songbirds by [259,260], showing that disorders like stuttering can be induced in Zebra finch by modifying the gene critical for timing control. Interestingly, the induced changes did not affect the structure of individual syllables in the bird songs. This is consistent with the synchronization hypothesis in which tactile anchoring and target approximation are two separate mechanisms, the former relying crucially on feedback control, while the latter relying mainly on feedforward control [102,238,261].

Even with the right genetic disposition, not only the ability to control the key articulators, but also the pathway to the brainstem may need time to fully develop after birth before synchronization can be attempted. This could be why canonical babbling, and with it, the ability to produce syllables, starts to emerge only around 6 months after birth [262], and even the order of the appearance of lingual consonants involved in the babbling follows that of the development of the tactile receptors in the tongue [217].

In summary, the synchronization hypothesis proposed in this paper may encourage future studies to look for specific neural activities that correspond to target approximation, edge synchronization, and tactile anchoring, or to demonstrate their implausibility. Given that the hypothesis is about the control of timing in motor movements, its neural correlates should show sufficient sensitivity to the temporal aspects of speech production. There has been some progress in this direction [257,263]. Future studies, however, need to be more purposefully designed to generate sufficiently specific evidence to either confirm or reject the synchronization hypothesis.

## 5. Conclusions and Broader Implications

This paper has proposed a new hypothesis about the syllable, positing it as a synchronization mechanism that makes the central nervous control of multiple articulatory movements in speech production possible by eliminating most of the temporal degrees of freedom. The hypothesis postulates three specific mechanisms: *target approximation*, *edge synchronization*, and *tactile anchoring*, which work together to execute the syllable—a recurring synchronization of multiple articulatory movements.

The proposed model differs from previous models of the syllable in that it is highly explicit, which makes it easily falsifiable. It can be rejected or at least weakened if it is demonstrated that reducing degrees of freedom is unnecessary, reducing temporal degrees of freedom via synchronization does not adequately resolve the DOF problem or simply does not happen, the target approximation model is defective, DSSTA is implausible, or tactile feedback is absent or ineffective. 

Since its initial proposal in the form of the time–structure model of the syllable [114], various aspects of the synchronization model have received empirical support. Edge synchronization is supported by articulatory and acoustic evidence for pitch–syllable synchrony in Mandarin [188] and English [190], consonant–vowel synchrony in Mandarin and English [153,155,156,157], vowel–tone synchrony in Mandarin [192], and resyllabification in English [232]. The benefit of reducing degrees of freedom by edge synchronization as well as dimension-specific sequential target approximation is supported by computational simulation of tone learning [191] and the learning of English words [121]. The synchronization mechanism has therefore already received strong support, and its benefit has also been demonstrated by vocal learning simulations. The establishment of this synchronization mechanism, especially with the elaboration of the dimension-specific sequential target approximation (DSSTA), cf. Section 3.1.2 and Section 3.2.2, offers a coherent account of many unresolved phenomena of speech, including formant transitions, coarticulation, coarticulation resistance, undershoot and assimilation, locus and locus equation, resyllabification, asymmetry of syllable onset and coda, pre-low raising of pitch, etc. Finally, the importance of tactile feedback is shown by the adverse effect of topical oral anesthetics on intelligibility and speech rate [220].

The synchronization hypothesis may have a number of broader implications. (A) It suggests that it is likely that the emergence of syllable articulation makes it possible for human vocalization to be particulated into discrete consonants, vowels, and tones—units capable of making phonological contrasts, which can then be combined into words, phrases, and sentences. Given that chimpanzees and bonobos, our closest relatives, cannot be taught to speak, syllable articulation could be one of the most critical steps in human language evolution. (B) The recent modeling simulation of vocal learning [121] suggests that canonical babbling in the form of onset-synchronized syllable sequences that starts typically from 7 months old could be a prerequisite for vocal learning [264]. (C) The synchronization hypothesis, itself partially motivated by the problem of too many degrees of freedom [3], may have implications for motor control in general [265,266,267], as well as for robotics [267], as any complex motor movements, either natural or bionic, would have to face exactly the same problem. And (D) the hypothesis may also have implications for the understanding and diagnoses of speech disorders, especially those related to articulation. Different types of disorders may be due to difficulty with either target approximation, edge synchronization, or tactile anchoring.

## Figures and Tables

**Table 1:** Motor synchrony vs. entrainment.

Property	Motor Synchrony	Entrainment
Synchrony in a single cycle?	Yes	N/A
Speed of achieving synchrony	Immediate (1–2 cycles)	Many cycles
Similarity in natural frequency?	No	Yes
In-synch out-synch undulation?	No	Yes
Under central/shared control?	Yes	No

## Data Availability

Data sharing is not applicable to this article as no new data were created or analyzed in this study.
